# Patient-Specific Instruments for Forearm Sarcoma Resection and Allograft Reconstruction in Children: Results in 4 Cases

**DOI:** 10.1155/2022/7005629

**Published:** 2022-10-31

**Authors:** Amaury Paulmier, Mathieu Raad, Charles-Edouard Verhelle, Laurent Paul, Pierre-Louis Docquier

**Affiliations:** ^1^Cliniques Universitaires Saint-Luc, Service de Chirurgie Orthopédique et Traumatologique, 10,Avenue Hippocrate, Bruxelles B-1200, Belgium; ^2^3D-Side, Rue André Dumont, Mont-Saint-Guibert 5-1435, Belgium; ^3^Secteur des Sciences de La Santé, Institut de Recherche Expérimentale et Clinique, Neuro Musculo Skeletal Lab (NMSK), Université Catholique de Louvain, Avenue Mounier 53, Bruxelles B-1200, Belgium

## Abstract

For pediatric malignant bone tumors located in the limbs, limb salvage surgery is the gold standard, but it requires adequate resection margins to avoid local recurrence. Primitive bone sarcomas of the forearm (radius or ulna) are very rare and the reconstruction remains challenging. We describe a method to ensure minimal but adequate resection bone margins with precision in four consecutive patients with primitive bone sarcomas of the forearm. During the preoperative planning, magnetic resonance imaging (MRI) was used to delineate the tumor and the tumor volume was transferred to computerized tomography (CT) by image fusion. A patient-specific instrument (PSI) was manufactured by 3D printing to allow the surgeon to perform the surgical cuts precisely according to the preoperative planning. The first PSI was used for the resection of the tumor, which adopted a unique position at the bony surface. A second PSI was intended for the cutting of the bone allograft so that it fitted perfectly with the bone defect. In all four cases, the safe margin obtained into the bone was free of tumor (R0: microscopically margin-negative resection). The functional result was very good in all four patients. This limb salvage surgical technique can be applied in forearm bone sarcoma and improves surgical precision while maintaining satisfactory local tumor control. It can also reduce the surgical time and allow a stable osteosynthesis.

## 1. Introduction

Primary bone tumors are rare in the European population (<0.2% of malignant neoplasms registered in the EUROCARE (European Cancer Registry)), and their incidence is 3 per 1,000,000 inhabitants per year [1]. Osteosarcoma and Ewing's sarcoma are the main malignant primary bone tumors in children and young adults [1]. Boys are more frequently affected than girls [1]. The forearm bones (radius and ulna) are rarely affected by osteosarcomas and Ewing's sarcomas. More exactly, the localization for Ewing's sarcomas is only 0.3% for the radius and 1.0% for the ulna [2].

Advances in diagnostic and therapeutic techniques in bone tumors have enabled the development of limb salvage surgery, particularly in the pediatric population [3]. In the case of limb salvage surgery, inappropriate resection margins are associated with an increased local recurrence rate [4]. Therefore, it is imperative to have adequate margins (free of tumor) to obtain local control of the tumor and improve oncological outcomes. However, large resection margins involve significant bone defects with a significant impairment of the limb function [5].

This article discusses a new surgical technique to decrease the resection margins of these tumors by using a patient-specific instrument (PSI). The PSI helps to intraoperatively reproduce resection plans that are planned before surgery. They are based on computed tomography (CT) and magnetic resonance imaging (MRI) sequences of the patient. This technique also helps in the reconstruction of the limb in order to preserve the function of the limb. Particularly, in young patients, limb function is of major interest. A lasting reconstruction must be carried out because the life expectancy of these patients has greatly increased [5].

Different reconstruction techniques exist for such bone defects: reconstruction by bone allograft, vascularized autograft, endoprosthetic reconstruction, and reimplantation of the tumor bone segment after being devitalized [5–7].

The massive bone allograft reconstruction technique offers the advantages of tendon and ligament reattachment [8, 9]. Other advantages are the biological incorporation of the graft (at least partial) and preservation of growth cartilage and joint [8, 10]. The 5-year survival rate is 80% for massive allografts [11].

A high rate of complications is, however, associated with allograft reconstruction, such as allograft fractures, infections of the operatory site, and nonunion between the allograft and the host bone [10, 11].

Despite these drawbacks, intercalary reconstruction by allograft bone grafting should be favored for the upper limb [12, 13].

PSIs have already been used in pedicle screw insertion, hip resurfacing, total knee arthroplasty, and long bones corrective osteotomy with more accuracy [14, 15]. In surgical oncology, the use of the PSI concept has been developed as a new surgical technique for bone tumor resection, allowing the surgeon to perform the trajectories of the cutting tool around the tumor [16].

The use of PSIs on bone allografts has previously been described in tibial reconstructions [17] and pelvic reconstruction [18]. Little data are available in the literature on tumor resection of the forearm, but studies on pelvic and tibial tumors have shown that PSIs guarantee good resection margins while reducing the risk of local recurrence [17, 18].

The aim of this article is to detail the surgical management of forearm primary bone sarcoma in young patients who require resection/reconstruction by using a PSI.

## 2. Patients and Methods

### 2.1. Patient Series (Table 1)

Four patients were operated on for primary bone tumor of the forearm by tumor resection and allograft reconstruction with PSIs. The primary bone tumor was Ewing's sarcoma in 3 cases (one radius and two ulnae) and one telangiectatic osteosarcoma of the radius. One patient had pulmonary metastases and one patient had two skip metastases into the proximal radius.

At the time of the resection, the age of patients ranged from 7 years and 5 months to 16 years and 3 months. The radiological evaluation consisted of radiography, CT scan, MRI, and fluorodeoxyglucose PET (positron emission tomography)/CT. Neurological deficits were absent. The diagnosis was confirmed by a surgical biopsy in all cases. The patients received multiagent neoadjuvant and adjuvant chemotherapy, according to EurAmos, Euro-Ewing 99, Euro-Ewing 2008, and Euro-Ewing 2012 protocols.

### 2.2. Preoperative Planning

The imaging techniques (CT scan and MRI) used for tumor diagnosis were used for the planning: Brilliance 40 CT-scanner (Philips, the Netherlands; 0.5 mm spacing between slices, 1 mm slice thickness, 120 kV peak voltage, and 99 mA tube current) and 1.5 TNT Scan Intera MRI (Philips, the Netherlands, 4 mm spacing between slices, 3 mm slice thickness, 550 ms TR, and 14 ms TE).

**Figure 1:**
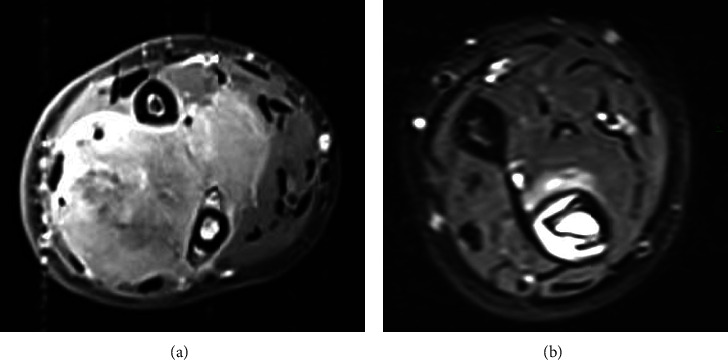
Patient 1 affected by voluminous Ewing's sarcoma of the ulna. After neoadjuvant chemotherapy, the tumoral volume had been decreased, allowing resection. (a) Prior to chemotherapy. (b) After chemotherapy.

Based on the prechemotherapy MRI sequences (Figure 1), the tumor was manually delineated by the surgeon by using a web-based segmentation tool (Customize V1; 3D-side). This software was developed by the 3D side and was used online by the surgeon. The tumor volume was drawn on each slice by using a simple polygon function. The tumor volume was automatically saved on the web server.

**Figure 2:**
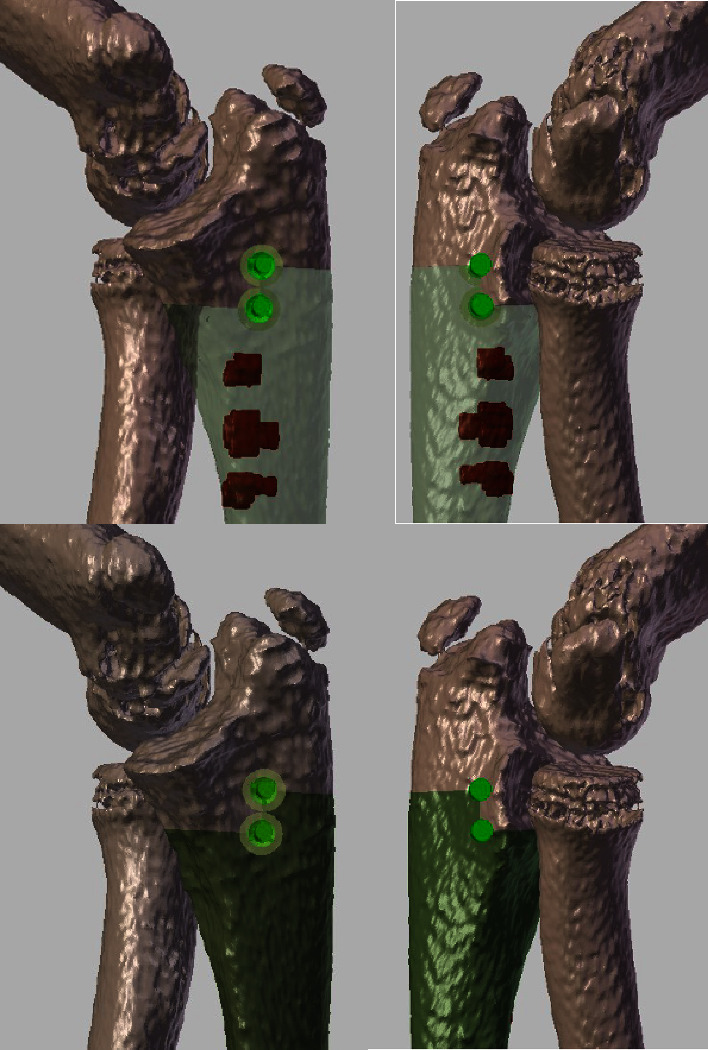
The same patient mentioned in Figure 1. Top left and right: the segmented tumor on MRI was merged with the CT scan. The tumor is in red (there were 3 small separated tumoral areas in the proximal ulna). The picture gives the detail of the preoperative planning for the proximal section. A step cut was chosen to improve stability. The chosen margin was 5 mm in order to preserve the joint. Bottom: the green zone in the ulna is the area that is planned to be resectioned. Left: medial view and right: lateral view.

By using a multimodal registration algorithm, MRI images and their associated tumor volume were merged with CT images. The tumor volume thus shared the same coordinate space as the CT. The CT image was segmented by using customized software to obtain a 3D representation of the bone. The tumor volume was converted into a 3D model. A combined 3D representation of the forearm bones and the tumor was therefore obtained (Figure 2). These 3D models were used to define the resection planes (target planes), which revealed the trajectories of the saw blade into the bone. The resection planes were first placed in contact with the tumor, and then translated with a safe margin defined by the surgeon. The usual safe margin was 10 mm, but in some cases, it could be decreased to preserve relevant structures such as physis or epiphysis.

### 2.3. Preoperative Planning for Allograft Cutting

The local bone bank scans all harvested allografts (SOMATOM Definition AS, Siemens; slice thickness of 0.35 mm, 0.7 mm spacing between slices, 120 kV peak voltage, and 99 mA tube current).

**Figure 3:**
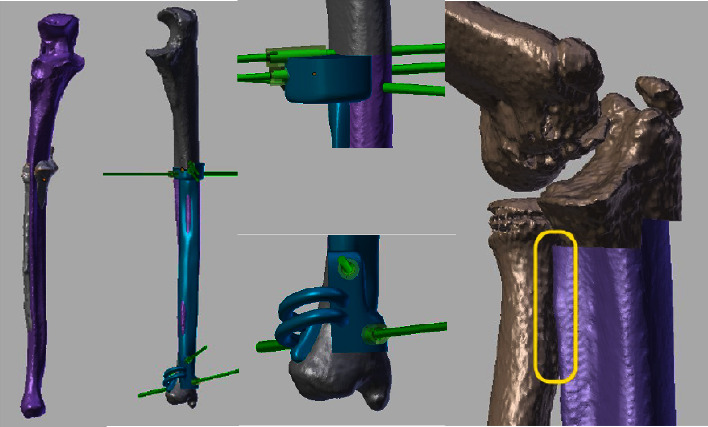
The same patient mentioned in Figure 1. The best matching allograft was obtained from an adult and was oversized. From left to right: (1) Ulna of the patient in grey and allograft in mauve. (2) PSI for allograft cutting. (3) Detail of the proximal part of the PSI and the distal part of the PSI. (4) Simulation of reconstruction with the allograft after tumor resection (patient in grey and allograft in mauve).

CT scans were sent to the 3D side for processing. After segmentation, the 3D models of all allografts were matched with the patient's bone to select the most suitable radius or ulna allografts (Figure 3). The objective was to find the best-fitting allograft for the postresection bone defect. For radius osteochondral allografts, particular attention was paid to the articular surface of the radial head. The resection plans were applied to the matched allograft. This process allowed us to obtain an identical cutting plan for the patient and the allograft and therefore, an optimal filling of the bone defect.

### 2.4. Patient-Specific Instruments

**Figure 4:**
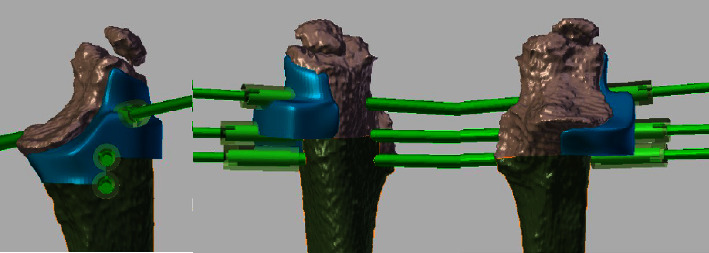
The same patient mentioned in Figure 1. The PSI for allograft cutting is placed at the medial side of the proximal ulna and is fixed by K-wires. It allows performing the step cut according to the planning. Left: medial view; middle: posterior view; right: anterior view.

Two PSIs were created for each patient: one for tumor resection and a second for allograft cutting. The PSIs were initially modeled by software (Blender 2.63.11). Each PSI was designed to accommodate a unique bone surface position (Figure 4). They held stainless steel cylinders to insert 2 mm Kirschner wires (K-wires) that fix the guide on the bone surface. The PSI was equipped with a flat surface representing the resection planes, guiding the saw blade during osteotomies.

The PSI was manufactured in a biocompatible material (polyamide 2200) by the technique known as selective laser sintering (SLS). It is a layer-by-layer additive manufacturing process. A laser sinters free powder by drawing a 2D shape. A new layer of powder is spread before drawing a second 2D shape. The process is repeated until the final 3D object is obtained.

The minimal time for virtual creation and for 3D printing of the guides is 15 days. However, the time is not critical due to the time of the presurgical chemotherapy in Ewing's sarcoma and osteosarcoma (several months).

### 2.5. Assisted Surgery

**Figure 5:**
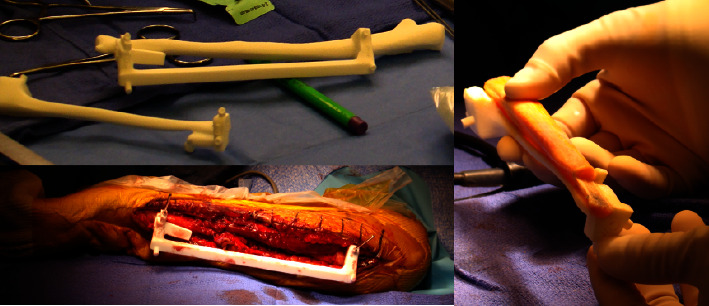
Patient 4 affected by a diaphyseal Ewing's sarcoma of the ulna. Left top: the tridimensional model of the patient ulna, the PSI for tumor cutting, and the PSI for allograft cutting are on the surgical table. Left bottom: the PSI for tumor resection is fixed to the patient's ulna. Right: the allograft and the PSI for allograft cutting.

During the surgery, the patients were placed in the decubitus position with an arm table. An anterior surgical approach (Henry's approach) was used in the two patients with radius sarcoma and a direct ulnar approach in the two patients with ulnar sarcoma (Figure 5).

The biopsy site was excised as an ellipse with the tumor. A progressive soft tissue dissection was performed to isolate the tumor with the expected margin.

The two PSI (one for the patient, one for the allograft) were previously sterilized by standard autoclave the day before the operation. Once the PSI has been placed in a unique position, it is fixed using K-wires. Using a surgical saw, the osteotomy is then performed with the chosen safety margin. Resection lengths ranged from 14.0 cm to 15.7 cm.

**Figure 6:**
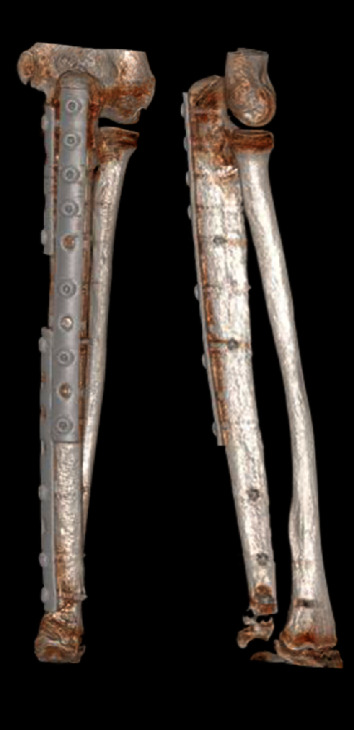
The same patient mentioned in Figure 1. CT scan performed 20 months after the surgery showing good healing at the proximal host-allograft junction and preservation of the distal ulnar physis.

Extemporaneous pathology analyses were performed on the distal and proximal resection sites to ensure adequate margins (free of tumors). After confirmation of a healthy margin, a reconstruction was undertaken. The second PSI was fixed to the allograft, taking into account the cutting thickness of the saw blade (1.5 mm). The adjusted allograft was placed on the bone defect and osteosynthesis was performed using plates and screws (Figure 6).

A long arm cast was put in place for all patients for 6 weeks.

### 2.6. Functional Evaluation

The functional outcome was evaluated by using the Musculoskeletal Tumor Society (MSTS) scoring system [19]. This functional classification system is scored out of 30 points established by the MSTS. This classification takes into account function, emotional acceptance, pain, hand positioning, dexterity, and lifting ability. Each variable is rated on a scale ranging from 0 to 5 points.

## 3. Results

The average surgical time was 176 minutes from the time of skin incision to the end of skin closure. Histological examination of the removed sarcoma confirmed the diagnosis of Ewing's sarcoma in three patients and telangiectatic osteosarcoma in one patient.

### 3.1. Obtained Margins

All the planned margins were obtained with less than 3 mm of error. All bone resection margins were assessed R0. All soft tissue resection margins were also R0, except one patient for whom the resection margin was R1 in the proximal radius. This patient required additional adjuvant radiotherapy. One patient developed a tumor recurrence within the soft tissue (nodule located in the flexor carpi ulnaris muscle requiring resection of this nodule 22 months postoperatively). There was no local recurrence into the bone for none of the patients. Physiotherapy was authorized after 6 weeks of cast immobilization for all patients.

### 3.2. Reconstruction Result

We used the classical radiograph, MRI, and PET/CT performed in the follow-up to assess the host-graft union. The results of the postoperative radiograph, CT, and MRI revealed a satisfactory host-graft contact, except in one patient. This last patient developed pseudarthrosis at the level of the proximal junction of the allograft and needed additional autologous bone graft at 12 months postoperatively (cancellous bone grafts harvested in the right iliac crest). For the other three patients, the radiological union was obtained at the graft-host junction at 6, 9, and 12 months.

Patient 2, after initial graft-host junction healing, sustained 2 successive allograft fractures, needing osteosynthesis the first time and finally allograft replacement with final graft-host junction healing.

### 3.3. Functional Result

Three patients had an MSTS score of 30/30 and one patient had an MSTS score of 23/30. This gives an average of 28/30 for all of our patients.

## 4. Discussion

This article reports the use of PSI for forearm bone sarcoma resection and reconstruction in children. PSI assistance is used for tumor resection and for cutting the massive allograft, allowing optimal reconstruction. We applied this technique to our 4 patients.

Surgical excision of the tumor requires obtaining a wide margin to avoid local recurrences. However, limb salvage surgery requires the preservation of a functional limb at the expense of obtaining safe margins [4]. The precise preoperative localization of the tumor allows precise planning, and the PSI improves the precision of the resection during the surgery. The combination of these techniques allows resection with adequate (free of tumor) but minimal safe margins, thus avoiding unnecessary resection and preserving, if necessary, articular cartilage in young patients. We generally plan a minimal margin of 10 mm, but we sometimes decrease it to 5 mm if needed for physis or joint sparing.

In the specific anatomic location of the forearm, one of the challenges was the relatively smooth surfaces of the radius and ulna. Given the triangular section of the radius and ulna, it was necessary to have a PSI with a surface straddling a crest of the bone to increase the stability of the PSI. Moreover, the proximal PSI and the distal PSI were connected by a bar. The fact that they were attached to each other further increased the precision in the positioning of the PSI. Another challenge was the size of the bones in our youngest patients (7, 9, and 10 years, respectively) and the need to respect the anatomical radial curves (supinator and pronator curvatures) which are important to keep the pronation and supination movements of the forearm. The most corresponding allograft was chosen in all cases respecting this radial curvature and the size of the bone as much as possible. The surgical approach was, in all cases, communicated to the engineer, and the PSI was created to be positioned according to the most accessible side of the bone communicated by the surgeon. Thanks to these precautions, during the surgery, no positioning problem was encountered nor was there conflict between PSI and the soft tissues.

In our series, we obtained adequate bone margins in the 4 cases (R0: microscopically margin-negative resection). The R1 margin was in the soft tissue in only one case.

Primary bone tumors localized into the forearm are extremely rare. A structural or functional deficit of an upper limb can significantly affect the quality of life.

The reconstruction by allograft of the forearm is extremely poorly published in the literature. Different techniques have been described such as the olecranonization of the radius initially described by Rydholm and then by Duncan [20, 21] or even a fibula autograft in the event of radial damage [22].

The absence of precise correction, after osteotomy, can lead to poor clinical results, especially in the upper limbs, where the anatomical configuration of the bones is of considerable importance for the function [23].

This is why the PSI technique allows the allograft to be cut with a high degree of precision, producing a graft of the optimal shape to fill the bone defect. The operating time is decreased as all the planning is carried out before the surgery.

Precise tumor and allograft sections obtained by the PSI yield a strong contact at host-graft junctions, resulting in stable osteosynthesis. Mechanical stability of the graft facilitates improved and faster bone healing and fusion due to increased blood vessel growth in the graft [24].

Open communication between surgeon and engineer is important. The engineer must have a strong clinical experience to understand the medical context and the prerequisites of the PSI that will be generated. The PSI should be localized to a single site on the bone surface that will be exposed without adding additional surgical approaches, unnecessary skin incisions, or dissection. Surgeons will be asked to anticipate the constraints of the surgery (for example, surgical approach, access to the bone surface, and presence of soft tissues). The engineer must inspect these clinical data and determine a compromise between the invasive character of the PSI and its stability on the bone surface. In our series of patients, precise positioning of the instruments was easily achieved in each case and the instruments were stable.

The preliminary results obtained from our four patients indicate that this technique is a clinically reliable method. Allograft reconstruction of the forearm can be performed with precision by using the PSI.

Other publications have already shown a decrease in local recurrence rate [18] by using the PSI in bone sarcoma resection and a decrease in operative time.

## 5. Conclusions

Limb preservation is the rule in oncological surgery. A structural or functional deficit of an upper limb can significantly affect the quality of life. For upper extremity sarcomas, the surgical team must find a balance between sufficient resection margins and reliable and durable reconstruction. Given the spatial constraints of the child's anatomy, it is impossible to obtain margins of several centimeters around the tumor. The techniques described in this article may help improve patient safety and surgical precision for the resection of forearm bone sarcomas and the accompanying reconstruction at these sites [26].

## Figures and Tables

**Table 1:** Details of the 4 patients.

	Case 1	Case 2	Case 3	Case 4
*Patient characteristics*				
Age (years)	10	7.4	9.3	16.2
Sex	F	F	F	F
Sarcoma type	EWS	EWS	Telangiectasic OS	EWS
Sarcoma localization	Ulna	Radius	Radius	Ulna
Metastasis	Lung metastases	No	2 skip metastases in the radius	No

*Surgical characteristics*				
Resection length (cm)	15.3	15.7	14.2	15.0
Resection type	Diaphyseal	Diaphyseal and proximal epiphysis	Diaphyseal and distal epiphysis	Diaphyseal
Preservation	Proximal and distal physes and epiphyses	Distal physis and epiphysis	Proximal physis and epiphysis	Proximal and distal epiphyses
Allograft reconstruction type	Intercalary allograft	Osteochondral allograft	Osteochondral allograft	Intercalary allograft
Operative time (minutes)	160	244	196	105

*Resection margin evaluation*				
Bone	R0	R0	R0	R0
Soft tissue	R0	R1 (in the soft tissue of the proximal section) needing adjuvant radiotherapy	R0	R0

*Complications and additional surgeries*				
Local complication	Skin necrosis needing scar revision (at 2 postoperative months)	-Skin necrosis needing scar revision (at 2 postoperative weeks)	None	Pseudarthrosis at proximal allograft-host junction needing autologous bone graft (at 13 postoperative months)
		-Allograft fracture needing plate osteosynthesis at 25 postoperative months)		
		-Allograft fracture needing allograft replacement at 40 postoperative months)		
Local recurrence	—	—	—	Local recurrence in the soft tissue (nodule in the flexor carpi ulnaris) needing nodule resection (at 22 postoperative months)

*Final result*				
Follow-up	2 years and 6 months	6 years	5 years and 11 months	9 years and 6 months
Final status of the patient	Alive and free of disease	Alive and free of disease	Alive and free of disease	Alive and free of disease
Allograft-host junction status	Healed	Healed	Healed	Healed
MSTS functional score	30/30	23/30	30/30	30/30
Final functional result	Excellent	Satisfactory	Excellent	Excellent

(EWS = Ewing's sarcoma; OS = osteosarcoma; *F* = female).

## Data Availability

Underlying data can be obtained upon request from the corresponding author.
